# Abstract of Meteorological Observations for March, 1855, Made at Philadelphia, Pa.

**Published:** 1855-05

**Authors:** James A. Kirkpatrick


*Abstract of Meteorological Observations for March,* 1855, *made at Philadelphia, Pa. Latitude* 39° 57′ 28″ *N., Longitude* 75° 10′ 40″ *W. from Greenwich.* By Prof. James A. Kirkpatrick.

| 1855. March. | Barometer. Daily Mean | Barometer. Mean Daily Regn. | Thermom. Daily Mean | Thermom. Mean Daily Range | Dew Point 2 P M. | Force of Vapor 2 P.M. | Rel. Humid 2 P. M. | Rain and melted Snow. | Prevailing Winds. | Remarks. |
|---|---|---|---|---|---|---|---|---|---|---|
| | Inches. | Inches. | Deg. | Deg. | Deg. | Inches. | Hunds. | Inch. | Points. | |
| 1 | 30.250 | .115 | 25.0 | 1.8 | 20.7 | 0.094 | 0.56 | | NW. | Cl'r. *Bar. high't* 30.272. *Th.* |
| 2 | 30.136 | .114 | 34.7 | 9.7 | 31.3 | .164 | .58 | | S. | Clear. [*lowest* 16°. |
| 3 | 29.854 | .282 | 39.3 | 5.0 | 28.0 | .129 | .37 | | Var.) | Cloudy. |
| 4 | 29.969 | .127 | 43.3 | 3.7 | 30.3 | .152 | .44 | | W. | M. cloudy; aft. and ev. clear. |
| 5 | 29.751 | .241 | 46.7 | 4.7 | 37.7 | .231 | .56 | | SW. | Cloudy. |
| 6 | 29.604 | .212 | 52.5 | 5.8 | 28.7 | .141 | .28 | | WNW. | Cloudy. |
| 7 | 29.705 | .101 | 37.8 | 14.7 | 29.3 | .147 | .57 | | N. | M. and aft. cloudy; ev. clear. |
| 8 | 29.845 | .141 | 36.0 | 2.2 | 20.0 | .084 | .34 | 0.005 | NW. | Clear. Rain during night |
| 9 | 29.558 | .287 | 41.5 | 5.5 | 37.3 | .223 | .64 | | NW. | Cloudy. |
| 10 | 29.859 | .301 | 29.0 | 12.5 | 21.3 | .095 | .49 | | NW. | Clear. [ring night. |
| 11 | 29.769 | .104 | 37.7 | 8.7 | 23.3 | .092 | .35 | 0.023 | SW. | Cloudy. *Snow or hail* du- |
| 12 | 29.781 | .134 | 37.3 | 3.7 | 34.3 | .194 | .82 | | NE. | Cloudy. |
| 13 | 29.738 | .205 | 36.7 | 2.0 | 34.7 | .199 | .90 | 0.751 | NE. | *Raining* all day. |
| 14 | 29.796 | .172 | 40.0 | 3.3 | 37.3 | .221 | .83 | | NE. | Cloudy. [bet. 7 & 8 A.M. |
| 15 | 29.641 | .155 | 44.7 | 4.7 | 44.7 | .299 | .92 | 0.645 | N. | M. rain; thunder & lightn'g. |
| 16 | 29.908 | .271 | 43.3 | 2.7 | 34.7 | .195 | .68 | 0.012 | N. | M. drizzling; aft. & ev. cl'dy. |
| 17 | 29.767 | .142 | 40.7 | 2.7 | 38.7 | .234 | .91 | 0.536 | NE. | *Raining* all day. |
| 18 | 29.917 | .167 | 40.7 | 1.3 | 29.3 | .147 | .57 | | NW. | Cloudy. |
| 19 | 29.937 | .085 | 39.3 | 5.3 | 30.7 | .155 | .48 | | SW. | Clear. |
| 20 | 29.916 | .080 | 36.3 | 5.0 | 24.0 | .103 | .44 | | NW. | M. and aft. cloudy; ev. clear. |
| 21 | 30.084 | .168 | 32.0 | 5.0 | 22.0 | .093 | .46 | | WNW. | Clear. |
| 22 | 29.936 | .148 | 32.8 | 2.5 | 22.0 | .093 | .46 | | (Var.) | M. and aft. cloudy; ev. clear. |
| 23 | 29.622 | .313 | 36.7 | 5.2 | 21.0 | .071 | .27 | | SW. | M. clear; aft. and ev. cloudy. |
| 24 | 29.412 | .270 | 39.0 | 8.3 | 35.3 | .201 | .62 | | NW. | Cl'dy; aft. sn'w. *B. l'st* 29.310. |
| 25 | 29.689 | .277 | 31.3 | 9.0 | 22.0 | .093 | .46 | | W. | M. and aft. cloudy; ev. clear. |
| 26 | 29.542 | .170 | 41.0 | 9.7 | 30.3 | .152 | .44 | 0.007 | SW. | Cloudy; evening *Rain.* |
| 27 | 29.608 | .192 | 37.3 | 9.0 | 25.0 | .110 | .46 | | NW. | Cloudy; aft. light Snow. |
| 28 | 29.781 | .173 | 32.3 | 5.0 | 23.0 | ·100 | .48 | | W. | M. clear; aft. *Snow.* |
| 29 | 29.826 | .045 | 38.2 | 5.8 | 30.3 | .155 | .57 | | W. | Clear. |
| 30 | 29.859 | .034 | 47.7 | 9.5 | 27.7 | .130 | ·26 | | SW. | Clear. |
| 31 | 29.690 | .169 | 52.8 | 5.2 | 33.7 | .203 | .34 | | (Var.) | Cloudy. *Therm. highest* 64°. |
| Means for March, 1855 | 29.798 | .174 | 38.8 | 5.8 | 29.3 | .152 | .53 | 1.97[illegible] | N. 60° W., 61–100. | |
| 4 yrs | 29.853 | | 41.5 | | 33.1 | | | 2.5[illegible]4 | N. 65½° W. 52–100. | |

The Monthly Range of the Barometer was 0.962 of an inch., and of the Thermometer 48°.

*Dear Sir,*—Two or three typographical errors occur in the Report for February, p. 256 of the Examiner.

The *Dew Point* on the 6th was —2.0, or 2 degrees *below* zero. In the remarks opposite the 7th, the *lowest of the Thermometer* was —1.0, or 1° *below* zero. In the column of *prevailing winds*, there should be no point between the letters; the direction for the 4th, 9th, 11th, 15th and 18th should be respectively WNW., NNW., WSW., WNW., and WNW., instead of W.NW., &c. J. A. K.

